# Histopathological Scoring Systems for Experimental Liver Injury Models

**DOI:** 10.7759/cureus.112930

**Published:** 2026-07-18

**Authors:** Tuba Yalcin

**Affiliations:** 1 Histology and Embryology, Batman University, Batman, TUR

**Keywords:** histological damage score, histopathological scoring, liver injury scoring, scoring systems, semi-quantitative assessment

## Abstract

Histopathological evaluation is a fundamental component of experimental liver injury studies because it allows direct assessment of structural alterations occurring at the tissue level. However, purely descriptive microscopic examination may be limited by observer-dependent interpretation and reduced comparability between experimental groups. For this reason, semi-quantitative scoring systems are widely used to convert morphological findings into numerical data and to improve the objectivity, reproducibility, and interpretability of histological assessment. In experimental liver injury models, commonly evaluated parameters include hepatocellular necrosis, inflammatory cell infiltration, hepatocyte degeneration or vacuolization, sinusoidal congestion or dilatation, steatosis, cholestasis, bile duct proliferation, fibrosis, and collagen deposition. The selection of scoring parameters should be guided by the type of experimental model, the expected pattern of injury, and the main objective of the study. In addition to conventional scoring, morphometric and digital image analysis methods may support histopathological evaluation by providing more objective measurements of specific tissue changes, particularly fibrosis, collagen accumulation, necrotic areas, steatosis, and immunohistochemical staining. Standardization of magnification, field selection, number of evaluated areas, staining conditions, scoring criteria, and blinded assessment is essential for obtaining reliable and comparable results. This review summarizes the main histopathological scoring approaches used in experimental liver injury models and emphasizes the importance of selecting appropriate, standardized, and reproducible scoring methods according to the specific characteristics of each model. Although this review focused on experimental liver damage models, standardized histopathological scoring can also support the interpretation of liver pathology in broader research contexts.

## Introduction and background

Experimental liver injury models, including models of hepatotoxicity, inflammation, steatosis, cholestasis, ischemia-reperfusion injury, and fibrosis, have been widely used to study the pathological processes involved in liver diseases. These models may show several characteristic histopathological findings, such as inflammatory cell infiltration, which refers to the accumulation of immune cells in damaged liver tissue; sinusoidal congestion or dilatation, which indicates impaired hepatic microcirculation and widening of sinusoidal spaces; steatosis, defined as excessive fat accumulation in hepatocytes; cholestasis, which reflects impaired bile flow or bile accumulation within the liver; and fibrosis, characterized by excessive extracellular matrix and collagen deposition.

Histopathological changes in the experimental liver injury models are usually assessed by histopathological examination of liver tissues, which directly shows various morphological changes, including necrosis, infiltration of inflammatory cells, hepatocytic degeneration, congestion of sinusoids, and steatosis, cholestasis, and fibrosis [1-3]. Qualitative histopathological assessment in experimental settings is typically subject to observer-dependent variability. To obviate this problem and make assessment of histological changes more quantitative, scoring of histopathological findings by means of predefined criteria is recommended and commonly performed. In this review, we describe the most frequently used scoring parameters for the respective types of liver damage in experimental animal models and discuss the issue of standardization of the respective scoring approaches.

## Review

The importance of quantitative measurement

Scientific research methods express an event, or any fact related to the issue under study, or characteristics of the subject of study, or variables affecting said subject numerically in the process of quantitative measurement. What is described in the qualitative study with expressions of "low," "moderate," "high," etc., in assessing any situation, phenomenon, or information, can be measured quantitatively and converted to numbers in the process of quantitative measurement. In this way, data collected in the process of quantitative measurement is more objective, comparable, and can be analyzed in a scientific manner. For this reason, the issue of validity and reliability of the measurement and assessment tools used in scientific research is very important and forms the basis of the results obtained [4].

Objective

In qualitative assessment, observations are often expressed in words. They can be described as low, moderate, high, good, poor, or marked. However, quantitative measurement of an event or of a condition expresses its quantity by means of figures in the form of numbers. This form of expression in numbers of the amount of a variable and of changes to it is more objective than words and can be compared. It minimizes the bias on the part of the researcher due to his/her personal views and makes the assessment more standardized. The objective of measurement is expressed by the terms validity and reliability. Validity of a quantitative measurement refers to the degree to which the used measurement tool really measures what it was intended to measure [4]. The term reliability refers to the extent to which the used quantitative measurement method or tool yields the same results under equal conditions [4].

The use of quantitative data in scientific research also facilitates comparison of events, conditions, or characteristics that have been measured in the same way over time or between different groups or under different circumstances. Thus, while the qualitative researcher notes that something has increased or decreased, the quantitative researcher can measure the extent of that change. Quantitative data collected as part of scientific research therefore not only describe the key findings from a research study, but can also be analyzed statistically in order to test hypotheses or to determine whether any differences identified are statistically significant or simply the result of chance. Another important point with respect to quantitative data and qualitative data is the reproducibility or reliability of data collected and/or analyzed within a study and/or the studies of other researchers. In many cases, studies support general conclusions that have been derived from earlier research studies. However, it is through replication that the findings of earlier research studies are verified, and it is only through the use of clearly defined, reliable, and valid quantitative data or procedures that replication of quantitative research is possible. In this way, other researchers are able to apply the methods that have been used by the researcher who conducted and/or analyzed the study, thereby allowing for replication [5].

Quantitative measurement is also an aid for evidence-based decision-making. In all fields of education, health, economy, management, and social sciences, decisions are taken on the basis of more than mere observation or subjective assessment. Measurable data as a basis for decision-making is monitored and evaluated. Quantitative measurement transforms data collected during research and evaluation into numbers that can be used to assess, compare, etc., in an objective, reliable, valid, and reproducible manner. Therefore, in scientific research, quantitative measurement is not only a means of collecting data, but it is also a tool for ensuring that findings from research and evaluation are scientifically sound [5].

In the context of experimental liver injury models, reliability and validity should not be considered only as classical methodological concepts, but also as essential principles for reproducible histopathological scoring. Reliable scoring requires that similar results can be obtained when the same tissue sections are evaluated under comparable conditions, whereas valid scoring requires that the selected parameters truly reflect the type and severity of liver injury being investigated. These principles are particularly important in semi-quantitative histopathological evaluation, where observer interpretation, magnification, field selection, staining quality, and scoring criteria may influence the final results. Therefore, the use of predefined scoring criteria, standardized microscopic fields, blinded evaluation, and, when appropriate, morphometric or digital image analysis may strengthen the objectivity and reproducibility of histopathological assessment. Although the concepts of reliability and validity are traditional methodological principles, their application remains highly relevant in current experimental liver pathology, especially with the increasing use of digital image analysis and quantitative tissue assessment.

Histology and Morphometric Measurements

Morphometry, or the quantitative measurement of certain features in histological sections, allows for an objective assessment of corresponding changes in liver tissue. Thus, for example, a simple increase in the average diameter of hepatocytes can be determined, as well as a higher average nuclear to cytoplasmic ratio. In addition, an increase in the area of sinusoids, an increase in the average diameter of the central vein or portal tracts, the average of necrotic area, the average of steatotic area, the average of inflammatory cell infiltrate, the average of fibrotic area, and the average of immunohistochemical positivity can all be quantified. As a result, instead of simple “yes/no” or semi-quantitative descriptions of changes in histological sections (e.g., as used for scoring fibrosis), results can be expressed in terms of area, length, or number and thus provide a solid statistical basis for comparing data obtained with the use of different experimental models and for evaluating dose-effect relationships. Morphometry is used to quantitatively determine the size and structural characteristics of cells and tissues in histological sections. In order to ascertain changes in liver tissue, quantitative determination, in addition to histological assessment of changes, is possible and indicates the extent and intensity of certain changes by specific values, for example, by areas, by lengths, or by densities [6,7].

In experimental liver injury studies, a special interest has to be put on fibrosis. Fibrosis means an increase in the extracellular matrix. The semi-quantitative scoring of fibrosis is limited by natural constraints. A quantitative assessment of the amount of fibrosis in a liver sample can be performed by determination of the collagen-positive area by using Sirius red or Masson’s trichrome staining and digital image analysis. For the computer-assisted digital image analysis of liver fibrosis, the collagen proportionate area (CPA) is an important parameter. Quantitative assessment of fibrosis by image analysis correlates with semi-quantitative scoring systems [8]. In addition to the assessment of the degree of fibrosis, the distribution and the structural constitution of fibrotic fibers can be analyzed by quantitative assessment by digital analysis of Picrosirius red-stained liver sections [9]. To validate the histological findings from the histological evaluation of experimental liver injury models, obtained from animal studies, it is essential to correlate them with those obtained from the histological scoring of the tissues. In rodent models of liver fibrosis induced by thioacetamide (TAA), carbon tetrachloride (CCl₄), or bile duct ligation (BDL), a number of studies have utilized automated and semi-automated image analysis methods for the quantitative assessment of fibrosis. Automated whole-slide image analysis for the quantification of fibrosis in preclinical models has the potential to offer increased sensitivity and to reduce inter-observer variability associated with the conventional scoring systems [10].

In addition, in models of steatosis, various parameters, such as the proportion of areas of fat-filled liver cells, the degree of hepatocellular changes by hepatocellular ballooning, the degree of lobular inflammation, and areas of necrosis, can be measured. Thus, in chronic liver diseases of non-alcoholic origin, including nonalcoholic fatty liver disease (NAFLD) and nonalcoholic steatohepatitis (NASH), in addition to semi-quantitative histological scores for steatosis, lobular inflammation, and degree of hepatocellular changes by hepatocellular ballooning, the qualitative assessment of the extent of liver tissue changes by digital pathology and morphometry can provide more objective results. Large areas of tissue can be analyzed using whole slide imaging followed by computer-assisted analysis, which reduces the sampling error and leads to more accurate results. Morphometric measurement provides useful information also for the evaluation of immunohistochemical markers in tissue sections. Semi-quantitative (such as percentage of area of positive staining, number of positive cells, optical density) and quantitative (e.g., H-score) methods are commonly used to score the intensity of a specific marker in studied cells in relation to control samples. The use of color image analysis in order to determine the percentage of area of positive staining has been successfully described by Willemse et al. Most important are the assessment of staining intensity and of areas of positive staining in immunohistochemical images using computerized image analysis, which allows for assessment of inter-laboratory variation and optimization of results. The assessment using the above-described semi-quantitative methods has been described in detail by Chlipala et al. and Shu et al. [11-13].

In terms of the analysis of the images and resulting data, it is important to standardize the analysis process as much as possible. This can be done by ensuring that all images are analyzed at the same magnification and that a sufficient number of fields from each sample are analyzed. In addition, it is important to ensure that the imaging conditions are kept constant for all samples and that the threshold used for the analysis is applied consistently to all groups. In this way, any observer effects are kept to a minimum, and the data can be used for statistical purposes to compare the results between the different groups [6,7]. Parameters, such as the percentage of area of necrosis or fibrosis, can be normalized to the total area of the tissue in the sample and thus used.

Histology allows the assessment of structural alterations that occurred during an experiment, whereas morphometry supports the qualitative findings of histology by providing quantitative, numerical values that are much more robust for data analysis than qualitative histology. By using digital image analysis to determine specific parameters, such as fibrosis percentage, necrosis, steatosis, inflammation, and positive cells for a specific marker by immunohistochemistry (IHC), the histological scores obtained can be significantly supported, and thus the differences between groups can be established more reliably. Therefore, by integrating the histological analysis with morphometry of specific structures, this approach provides a powerful tool to both establish and to verify the structural and also the mechanistic changes induced by a specific treatment in an experimental model of liver injury.

Morphometric Measurements in Liver Tissue

Quantitative assessment of liver tissue by measurement of liver morphometry was performed by means of digital image analysis of liver histological sections. Morphometry of liver tissue is considered a quantitative analysis of the histological sections in order to obtain data on the structural components of the liver parenchyma. While qualitative assessment of the histologically evaluated liver tissue permits to describe the changes by means of descriptive terms such as increased, decreased, promoted etc., the changes of the liver tissue architecture are quantified by means of morphometry, and described by figures such as area, diameter, length, frequency, ratio, and in percent, etc. for an exact and for a reproducible assessment of the extent of liver injury [6,7]. For morphometric determination in the liver, different parameters can be measured. The diameter of single hepatocytes as well as of their nuclei, the nuclear-to-cytoplasmic ratio of hepatocytes, the area of single sinusoids, the diameter of the central veins as well as of the portal tracts, the amount of necrotic, steatotic, and fibrotic tissue in relation to the total amount of liver tissue, the CPA, and the rate of positive immunohistochemical reactions are typical parameters. Their quantitative determination gives a more exact description of liver tissue than simple descriptions of changes to the tissue structure, which are merely described by terms such as "increased," "decreased," "prominent," etc., and allows for a comparison of the effects of treatment as well as of liver injury in experimental studies. The parameters can be determined from single histological sections and from a sufficient number of sections from each experimental animal. Assessing fibrosis is of particular interest by means of morphometry. The process of fibrosis involves the increasing deposition of extracellular matrix by hepatic stellate cells (Ito cells) following liver injury. This accumulation of collagen and other connective tissue components results in an increase in fibrotic areas within the liver. The assessment of fibrosis by means of semi-quantitative scoring systems of histological sections has some limitations. A quantitative assessment of fibrosis can be performed by calculating the ratio of collagen-positive areas to the total area of the tissue section by means of digital image analysis after staining sections with Sirius red or Masson’s trichrome. The resulting ratio is referred to as CPA and is an important parameter for the quantitative assessment of fibrosis [8,14].

In fibrosis quantification, more continuous and thus more analytical data are provided by digital image analysis than by semi-quantitative scoring. In this context, Arjmand et al. used digital image analysis to determine the CPA in liver tissue as a quantitative parameter for evaluating hepatic fibrosis [14]. Courtoy et al. furthermore used Picrosirius red-stained sections for quantification of fibrosis by digital image analysis. They were able to assess not only the extent of fibrosis but also its distribution and the characteristics of single fibrotic structures in more detail [9]. Thus, in addition to the presence of fibrosis, its amount can be quantified by morphometry. Quantitative assessment of fibrosis in rodent models of fibrosis is widely used in experimental research. The number of fibrotic areas has been quantified in experimental models of fibrosis induced by TAA, CCl₄, and BDL by using automated or semi-automated analysis of images in whole-slide digital images, such as second-harmonic generation/two-photon excited fluorescence images obtained by non-invasive microscopy [15]. Using automated whole-slide image analysis of digital histopathological images, obtained from preclinical liver fibrosis models, provides more sensitive quantitative data than conventional semi-quantitative histological scoring systems [10]. Fibrosis is only one aspect of severe liver injury. In particular, models of toxic or acute liver damage can be analyzed with respect to the amount of necrotic areas, the density of inflammatory cells, the degree of dilatation of sinuses, the amount of congestion, and the various forms of degeneration of single hepatocytes. In various models of fatty liver disease and steatohepatitis (NASH), the amount of steatotic areas, the amount of the tissue occupied by the lipid-filled vacuoles, the degree of hepatocellular ballooning, and the degree of lobular inflammation can be analyzed. For an exact semi-quantitative scoring of the extent of steatosis, lobular inflammation, and various forms of hepatocellular changes, the Nonalcoholic Fatty Liver Disease Activity Score (NAS) and the corresponding scoring system for rodent models of NASH have been developed [2,16].

Quantitative and semi-quantitative assessment of specific protein expression in hepatocytes or extracellular liver tissue using IHC is an important component of experimental liver research. For most IHC markers, staining is assessed semi-quantitatively. The amount of the stained protein can be scored based on the intensity and the distribution of the staining in the liver tissue. By using IHC scoring systems, an objective assessment of the protein expression can be made. Digital image analysis methods, including color image analysis of the IHC-stained sections, can be used to measure the percentage of immunoreactive area, which represents the amount of the stained protein in the IHC sections. Digital image analysis can improve the objectivity and reproducibility of IHC staining assessment [11,12]. For quantitative assessment of morphometric characteristics in liver tissue, a standardised analysis is required. This also refers to the process of digital image analysis. The evaluation has to be performed under the same conditions. Thus, all images have to be taken at the same magnification. For the analysis of one animal, several fields have to be chosen randomly, and in each field, all parameters have to be measured. The conditions for taking images, especially the lighting and the camera, have to be kept constant for all samples. Furthermore, threshold values have to be set in an equal manner for all groups. To ensure reliable results, the evaluator should be blinded to group allocation. The same principles for guaranteeing reproducibility as are known for experiments with animals have to be followed for morphometric assessment [7]. Morphometry of the liver tissue thus transforms the usual qualitative evaluation of tissue structures into figures that can be worked with. The number of parameters measured in this way - such as percentage of fibrotic area, CPA, percentage of necrotic area, percentage of steatotic area, of inflammatory cells, of sinusoidal dilations and of congestion, and also the evaluation of immunohistochemical parameters - renders more solid the usual merely descriptive and qualitative evaluation of the alterations of the liver tissue, and thus morphometry of the liver also, combined with the corresponding histological evaluation, in experiments of liver injury is a very useful method that enables an adequate, specific, objective, and more accurate assessment of the various alterations of the liver and also of the degree of the studied injury, and at the same time supports comparison between the different study groups.

Methodology

The aim of this article is to review the histopathological scoring systems used in various experimental liver injury models. Therefore, this article is designed as a narrative methodological review and does not aim to serve as a systematic review or meta-analysis. The most frequently used histopathological scoring systems are summarized and discussed, particularly in terms of the parameters evaluated, model-specific applicability, morphometric assessment of liver tissue, and standardization of histopathological evaluation. A literature search was conducted using PubMed, Web of Science, Scopus, and Google Scholar. The search covered English publications available from April 1, 2026, to June 28, 2026, as many key scoring systems and methodological studies have been previously published and continue to be widely used in experimental liver injury research. Search terms related to experimental liver injury models were combined with terms related to histopathological scoring and semi-quantitative evaluation. The main search terms included “experimental liver injury,” “histopathological scoring,” “semi quantitative scoring,” “liver injury score,” “Suzuki score,” “NAFLD Activity Score,” “rodent liver fibrosis,” “acetaminophen-induced liver injury,” “carbon tetrachloride induced liver injury,” “thioacetamide-induced liver fibrosis,” “bile duct ligation,” “collagen ratio area,” “morphometric evaluation,” “digital image analysis,” and “immunohistochemical scoring.” Original experimental studies and review articles were considered suitable if they described experimental liver injury models, histopathological scoring criteria, morphometric or digital image analysis methods, or immunohistochemical scoring approaches related to the assessment of liver tissue damage. Additional relevant publications were identified by searching the reference lists of the selected articles. Priority was given to studies that clearly defined scoring criteria, described the histological parameters evaluated, or applied established scoring systems in experimental liver injury models.

Studies that were not related to liver tissue damage, did not include histopathological or morphometric evaluation, focused solely on clinical human liver disease without being related to experimental models, were published in languages ​​other than English, or lacked sufficient methodological detail regarding tissue evaluation were excluded. In keeping with the narrative design of this review, no formal risk assessment, meta-analysis, or systematic quality scoring was performed. The selected literature was synthesized descriptively to provide an overview of the application of histopathological scoring systems in experimental liver injury studies.

Hepatocellular Necrosis Scoring

Hepatocellular necrosis is the most commonly evaluated histopathological parameter in liver injury models, including N-acetyl-p-aminophenol (APAP)-, CCl₄-, and lipopolysaccharide/D-galactosamine (LPS/D-GalN)-induced liver injury models, and ischemia/reperfusion-induced liver injury. The extent, location, and severity of necrosis within the liver can serve as a criterion to assess the degree of liver damage [3,17]. For assessing necrosis, a semi-quantitative scoring system is used, i.e., a score of 0-4 or 0-5 is assigned. The absence of necrosis is scored as 0, while single-cell necrosis is scored as 1, and the rest of the conditions, such as focal necrosis, centrilobular or zonal necrosis, bridging necrosis, and massive necrosis, are scored on the basis of the extent of injury. In the Suzuki scoring system for hepatic ischemia-reperfusion injury, the score for parenchymal necrosis is given from 0 to 4. In this scoring system, the degree of sinusoidal congestion and hepatocyte vacuolization is also taken into account [1,17]. In case of APAP-induced liver injury, necrosis is characterized by involvement of hepatocytes around the central vein and can be scored on the basis of the extent of necrosis induced by the drug. The evaluation of necrosis is made by scoring randomly selected fields under similar magnification and blinded conditions. In the case of a blinded condition, the observer will not know the group to which the animal belongs (Tables 1, 2) [3,7,18-23].

**Table 1:** Histopathological scoring approaches used for the assessment of hepatocellular necrosis in experimental liver injury models. The table summarizes commonly used semi-quantitative scoring systems for hepatocellular necrosis in different experimental liver injury models, including hepatic ischemia-reperfusion injury, acetaminophen-induced liver injury, LPS/D-GalN-induced acute liver injury, and experimental acute liver failure models. The table indicates the scoring range used in each model and explains how necrotic changes are graded according to their extent and severity. APAP: acetaminophen/N-acetyl-p-aminophenol; LPS/D-GalN: lipopolysaccharide/D-galactosamine

Model/source	Necrosis scoring	Explanation
Hepatic ischemia-reperfusion model - Suzuki score	0-4	The Suzuki score is one of the most commonly used systems for diagnosing liver ischemia-reperfusion injury. Sinusoidal congestion, cytoplasmic vacuolization, and parenchymal necrosis are each evaluated separately on a scale of 0-4. For necrosis, 0 = none, and 4 = >60% parenchymal necrosis [1,18].
APA/acetaminophen model	0-5	In APAP-induced liver damage, hepatocellular damage is graded on a scale of 0-5. 0 represents a normal histological appearance, while 5 indicates the most severe hepatocellular damage. In the APAP model, necrosis is usually prominent in the centrilobular region [3].
Acute APAP liver injury model	0-3	Some APAP studies use a simpler 0-3 damage score. In this system, normal liver tissue is seen in the control or time 0 group, while damage is reported to increase 12-24 hours after APAP administration [19].
LPS/D-GalN acute liver injury model	Typically a semi-quantitative damage score.	The LPS/D-GalN model is commonly used to induce acute liver failure. Histologically, it shows widespread necrosis, inflammation, hemorrhage, and disruption of liver architecture. In this model, necrosis is mostly assessed within the total histological damage score [20,21].
Inflammation + necrosis combined scoring.	0-3	In some studies, inflammation and necrosis are scored together: 0 = no inflammation/necrosis, 1 = diffuse immune cells or mild necrosis <10%, 2 = significant necrosis 10-50%, 3 = diffuse inflammation or severe necrosis >50%. This system can be particularly useful in total liver lesion scores [6,22].
Modified Suzuki score	0-5	In some ischemia-reperfusion studies, the Suzuki score has been modified to evaluate necrosis on a scale of 0-5. In this modified system, a score of 5 may indicate very severe damage, such as >80% confluence necrosis [1].
Experimental acute liver failure models	Necrosis percentage/damage percentage	In acute liver failure models, necrosis can sometimes be presented as a percentage of necrotic area rather than a score. Reviews evaluating preclinical acute liver failure models have reported that hepatic necrosis rates vary depending on the duration of ischemia [23].

**Table 2:** General semi-quantitative scoring scale for hepatocellular necrosis in liver tissue sections. The table provides a general 0-5 grading system for evaluating hepatocellular necrosis in experimental liver injury. The score increases according to the severity and distribution of necrotic changes, ranging from the absence of necrosis to massive necrosis or loss of lobular architecture. This scoring scale may be adapted according to the specific experimental model and study design.

Scoring	Explanation
0	No necrosis
1	Single-cell necrosis or minimal focal necrosis
2	Mild necrosis; limited foci
3	Moderate zonal/centrilobular necrosis
4	Widespread and severe necrosis
5	Massive necrosis or loss of lobular architecture

Inflammatory Cell Infiltration

Assessment of inflammatory cell infiltration within the liver in experimental liver injury refers to the quantification of inflammatory cells in the liver parenchyma, in portal tracts, or in areas of necrosis. This parameter is assessed in many studies on liver injury by APAP, CCl₄, LPS/D-GalN, and ConA, in models of ischemia-reperfusion injury or in models of NASH/NALFD-induced liver injury. The inflammatory cell infiltration is scored based on the extent, density, and localization of the inflammatory cell infiltrate. In the NAS, lobular inflammation is scored from 0 to 3. In experimental models of toxic and acute inflammatory liver injury, a semi-quantitative scoring system of 0 to 4 is used (Table 3) [1,2,16].

**Table 3:** Semi-quantitative scoring system for inflammatory cell infiltration, hepatocyte degeneration, and hepatocyte vacuolization in experimental liver injury models. This table presents a combined 0-4 semi-quantitative scoring system for evaluating two major histopathological features of liver injury: inflammatory cell infiltration and hepatocyte degeneration/vacuolization. Inflammatory cell infiltration is scored according to the density, distribution, and extent of inflammatory cells within the hepatic parenchyma, portal areas, or perinecrotic regions. Hepatocyte degeneration and vacuolization are scored according to the severity of hepatocyte swelling, hydropic degeneration, cytoplasmic pallor, vacuole formation, and disruption of hepatic parenchymal architecture. This combined scoring approach may be used in toxic, inflammatory, ischemia-reperfusion, and metabolic liver injury models.

Scoring	Inflammatory cell infiltration	Hepatocyte degeneration and vacuolization
0	No inflammatory cell infiltration	Normal hepatocyte morphology
1	Mild, few, and scattered inflammatory cells	Mild hepatocyte swelling or a small number of cytoplasmic vacuoles
2	Focal clusters of inflammatory cells	Focal hepatocyte degeneration and limited vacuolization
3	Widespread and marked inflammatory cell infiltration	Widespread hydropic degeneration and marked hepatocyte vacuolization
4	Severe, diffuse inflammatory cell infiltration with disruption of tissue architecture	Severe diffuse hepatocyte degeneration, extensive vacuolization, and disruption of parenchymal architecture

Hepatocyte Degeneration and Vacuolization

Liver damage may be expressed by the degree of hepatocyte degeneration and vacuolization. The parameters used for their evaluation are hepatocyte swelling, cytoplasmic atrophy, and pallor, cytoplasmic and cellular hydropic degeneration, and granular change, as well as formation of cytoplasmic vacuoles. These changes are most frequently assessed in liver injuries caused by APAP, CCl_4_, in models of ischemia/reperfusion, and in experiments modeling NAFLD/NASH [1-3]. Suzuki score for vacuolization is another major scoring parameter used mainly for the assessment of ischemia-reperfusion injury [1]. Vacuolization in hepatocytes is also evaluated under the NAS, in which hepatocyte ballooning is scored from 0 to 2 for both zones [2]. Degree of hepatocyte degeneration and vacuolization can therefore be assessed using the vacuolization component of the Suzuki score and the overall scoring principle used for semi-quantitative scoring in tissue sections (Table 3) [1,6].

Sinusoidal Congestion and Dilatation

Histopathological indicators of disturbance of the hepatic microcirculation in experimental liver injury are sinusoidal congestion and dilatation. This type of liver damage can be investigated within the framework of models of toxic liver injury, of acute inflammatory liver injury, and of hepatic ischemia-reperfusion injury. Suzuki scoring system has been widely used for the assessment of various histopathological changes, including sinusoidal congestion, hepatocyte vacuolization, and necrosis of liver parenchyma. For evaluation of sinusoidal congestion, 0 points are given for no congestion, 1 point for minimal, 2 points for mild, 3 points for moderate, and 4 points for severe congestion of sinusoids. Suzuki scoring system has been used for the quantitative assessment of ischemia-reperfusion-induced liver injury (Table 4) [1,17,18,24].

**Table 4:** Semi-quantitative scoring system for sinusoidal congestion/dilatation, hepatic steatosis, and hepatocyte ballooning. The table summarizes the scoring of three common histopathological findings in experimental liver injury models. Sinusoidal congestion/dilatation is graded according to vascular changes and architectural disruption; hepatic steatosis according to the percentage of hepatocytes containing lipid droplets; and hepatocyte ballooning according to the number of ballooned hepatocytes.

Scoring	Sinusoidal congestion/dilatation	Hepatic steatosis	Hepatocyte ballooning
0	No sinusoidal congestion or dilatation	No steatosis or <5% of hepatocytes affected	No ballooning
1	Minimal congestion or mild sinusoidal dilatation	Mild steatosis; 5-33% of hepatocytes affected	Few ballooned hepatocytes
2	Mild to moderate congestion with pronounced sinusoidal dilatation	Moderate steatosis; 34-66% of hepatocytes affected	Significant or numerous ballooned hepatocytes
3	Moderate to severe congestion with widespread sinusoidal dilatation	Severe steatosis; >66% of hepatocytes affected	Not applicable
4	Severe and diffuse congestion/dilatation with disruption of parenchymal architecture	Not applicable	Not applicable

Steatosis and Hepatocyte Ballooning

Here, we discuss the important histological features, steatosis and hepatocyte ballooning, in animal models of NAFLD/NASH, high-fat diet, obesity, and diabetes, and in liver injury caused by alcohol or toxins. In histological assessment of NASH, the degrees of steatosis, lobular inflammation, and hepatocyte ballooning are assessed as the most important features of liver injury. The features of steatosis, lobular inflammation, and hepatocyte ballooning are scored from 0 to 3 and from 0 to 2, respectively, in the NAS. In rodent models of NAFLD, steatosis can be scored for the presence of both macrovesicular and microvesicular fat droplets in the liver cells, and hepatocellular changes can be scored for the degree of hepatocellular hypertrophy/apoptosis/ballooning. In the NAS for assessment of NAFLD samples, steatosis is scored from 0 to 3, and hepatocellular ballooning is scored from 0 to 2. Within rodent NAFLD models, parameters such as the degree of macrovesicular and microvesicular steatosis and hepatocellular hypertrophy/ballooning can be quantitatively assessed (Table 4) [2,25].

Scoring for Cholestasis and Bile Duct Proliferation

Cholestasis involves the uptake of bile pigments by hepatocytes, or in more severe cases by the bile canaliculi, in the event of an arrest in bile outflow. Bile duct proliferation, on the other hand, is typically seen in models of cholestatic liver injury, including BDL, drugs that cause cholestasis, and chronic biliary damage. In BDL models, in addition to the above parameters, cholestasis, portal inflammation, and fibrosis are also assessed (Table 5) [26-28].

**Table 5:** Semi-quantitative scoring system for cholestasis and bile duct proliferation in experimental liver injury models. The table summarizes the 0-4 scoring criteria for cholestasis and bile duct proliferation/ductular reaction in cholestatic and chronic biliary liver injury models. Cholestasis is graded according to bile pigment accumulation, bile plug formation, and parenchymal disruption. Bile duct proliferation is graded according to ductular reaction, portal expansion, associated inflammation, and biliary fibrosis.

Scoring	Cholestasis	Bile duct proliferation/ductular reaction
0	No cholestasis	No proliferation
1	Mild bile pigment accumulation	Mild ductular reaction
2	Moderate canalicular or hepatocellular bile accumulation	Moderate bile duct enlargement and portal dilation
3	Disseminated and overt cholestasis	Significant ductal proliferation, which may be accompanied by portal inflammation or fibrosis
4	Diffuse cholestasis, bile plugs, and parenchymal disruption	Severe and widespread ductal proliferation with significant biliary fibrosis

Fibrosis and Collagen Accumulation

Chronic liver fibrosis (chronic damage) is characterized by an excessive accumulation of extracellular matrix components, mainly collagen. Fibrosis can be evaluated in various experimental models of liver damage, such as CCl_4_, TAA, BDL, models of NAFLD/NASH, and models of alcoholic liver damage. Fibrosis can be scored by evaluating the degree of collagen accumulation in the liver tissue, by assessing the extent to which portal/periportal spaces are filled with collagen, and by the formation of septa. In addition, bridging fibrosis and cirrhosis can be evaluated. The three models, CCl_4_, TAA, and BDL, are the most commonly used rodent models for experimental fibrosis. For fibrosis evaluation, the general tissue structure can be evaluated by hematoxylin and eosin (H&E) staining. For collagen accumulation, specific stainings like Masson trichrome and Sirius red are preferred. Sirius red can especially be used to quantify types I and III collagen by digital image analysis. The ratio of the collagen-stained area to the total liver tissue area is expressed as the CPA. This parameter is important for quantitative fibrosis evaluation (Table 6) [9,10,14,15,29-31].

**Table 6:** Scoring systems for liver fibrosis and collagen accumulation in chronic experimental liver injury. The table summarizes two commonly used approaches for evaluating liver fibrosis. The general semi-quantitative fibrosis score grades collagen deposition, septal formation, bridging fibrosis, and cirrhotic remodeling on a 0-4 scale. The Ishak fibrosis staging system provides a more detailed 0-6 staging approach based on portal fibrotic expansion, bridging fibrosis, nodule formation, and cirrhosis.

Scoring	General fibrosis and collagen accumulation score	Ishak fibrosis staging system
0	No fibrosis	No fibrosis
1	Mild portal or periportal collagen deposition	Fibrotic expansion in some portal areas; no septa
2	Portal fibrosis and limited septal formation	Fibrotic expansion in most portal areas; no septa
3	Significant septal fibrosis or bridging fibrosis	Fibrotic expansion in most portal areas with rare portal-portal bridging
4	Extensive bridging fibrosis, nodular remodeling, or cirrhosis development	Fibrotic expansion in portal areas with marked portal-portal or portal-central bridging
5	Not applicable	Marked bridging fibrosis with nodule formation; incomplete cirrhosis
6	Not applicable	Cirrhosis with widespread nodular architecture and fibrotic septa

Total Histological Damage Score

Total histological damage score is given by the sum of all the individual parameters (necrosis, inflammation, degeneration, vacuolization, congestion, steatosis, and fibrosis) detected within the liver tissue. These parameters can be quantified in order to provide an overview of the severity of tissue damage [8]. In this study, a histopathological histoscore table was established to calculate histoscores by attributing score values of 0 to 4 (0: none; 1: 0-25%; 2: 26-45%; 3: 46-75%; 4: 76-100%) for each criterion (Table 7) [32,33].

**Table 7:** Scoring system for total histological damage and selected histopathological changes in liver tissue. The table summarizes a combined scoring approach for evaluating overall histological damage and selected liver tissue alterations. The total histological damage score reflects the general severity of liver injury, whereas individual parameters are graded according to the presence, severity, and localization of specific histopathological findings. Kupffer cells are resident macrophages of the liver.

Scoring	Total histological damage	Severity-based histopathological changes	Localization-based histopathological changes
0	No damage	No sinusoidal dilation, hepatocyte vacuolization, inflammatory foci, degenerated hepatocyte cords, or hemorrhagic areas	No hydropic degeneration
1	Minor damage	Mild sinusoidal dilation, hepatocyte vacuolization, inflammatory foci, degenerated hepatocyte cords, or hemorrhagic areas	Sinusoidal dilation around the central vein and portal region
2	Moderate damage	Moderate sinusoidal dilation, hepatocyte vacuolization, inflammatory foci, degenerated hepatocyte cords, or hemorrhagic areas	Inflammatory foci around and near the central vein and portal region
3	Significant damage	Severe sinusoidal dilation, hepatocyte vacuolization, inflammatory foci, degenerated hepatocyte cords, or hemorrhagic areas	Activated Kupffer cells around the central vein and portal region, and extending to more distant areas
4	Severe damage	Not applicable	Increased glycogen concentration in hepatocytes throughout the liver
5	Massive damage or loss of tissue architecture	Not applicable	Fibrosis or advanced architectural alteration

Scoring of Various Parameters in the Liver

Histopathological changes on the surface of the liver tissue samples were measured in 10 non-overlapping areas per sample under x10 magnification. The degree of sinusoidal dilatation, hepatocyte vacuolization, foci of inflammation, degenerated hepatocyte cord formations, and hemorrhagic areas was scored on a scale of 0-3, i.e., 0 for the absence of changes and 1 for slight, 2 for moderate, and 3 for severe changes. Histopathological damage scores were determined for each of the experimental animals using the above-mentioned parameters (Table 7) [34-37].

Calculation of Mean Lobule Area in the Liver

Liver tissue sections were evaluated under a light microscope at a magnification of ×40 after staining with HE. Randomly selected photos were taken, and in 10 lobules per animal, the corresponding areas were measured on the digital micrographs by using image analysis software [38].

The animals were treated as replicates (n = 8). The measured values represent the actual area in square micrometers (μm²). The mean hepatic lobule area can theoretically be calculated as a morphometric parameter in histopathological studies. However, the use of this parameter has limitations, especially in experimental animal studies. First, it is often difficult to clearly define the boundaries of a lobule in an animal liver. The boundaries between lobules are often not sharply defined. Second, the apparent relationship between the central vein and portal areas can be influenced by the plane of sectioning. The vascular diameter within a lobule can also appear to be narrower or wider, depending on the level and angle of sectioning. As a consequence, the mean hepatic lobule area is not a reliable parameter for histopathological assessment in experimental liver injury models. However, other parameters such as necrosis, inflammation, congestion/dilatation of sinusoids, steatosis, fibrosis, and the percentage of the collagen-positive area in the liver tissue are suitable for histopathological and morphometric assessment.

Determination of Average Central Vein Diameters in the Liver

**Figure 1:**
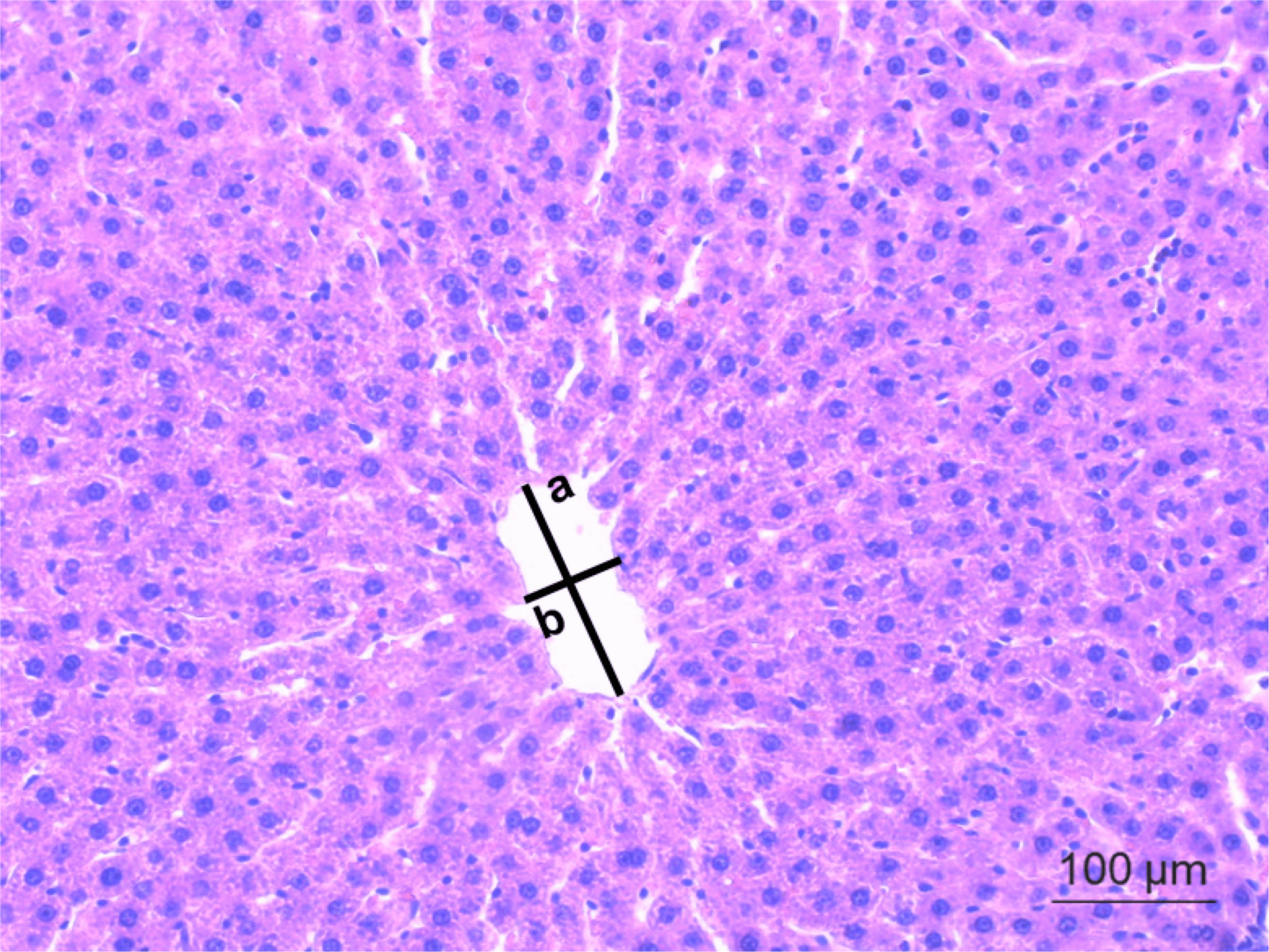
Morphometric measurement of the mean central vein diameter in H&E stained liver tissue. The figure shows a representative H&E-stained liver section used for morphometric assessment of the central vein. The long black line labeled "a" indicates the major diameter of the central vein, while the shorter perpendicular black line labeled "b" indicates the minor diameter passing through the midpoint of the major diameter. Measurements were performed on calibrated microscopic images and expressed in micrometers (µm). The mean central vein diameter was calculated from these two perpendicular measurements using the formula D = √(a × b), where D represents the mean central vein diameter, a represents the major diameter, and b represents the perpendicular minor diameter. Scale bar: 100 µm.

Diameters of the central vein were measured from images of the central vein taken using an image analysis program (Figure 1). In the program, the diameters of the central vein major (a) and minor (b) were measured in μm, and the average diameter of these values was determined using the following formula [38,39].

Determination of average central vein diameters in the liver is morphometrically possible, but has to be measured after prior standardization because the vein’s diameter changes depending on the plane, the fullness of the vein, on congestion, on tracking of the tissue and on the angle of the section. Therefore, it is more appropriate to present it as an auxiliary morphometric parameter rather than as the main histopathological scoring parameter.

The mean central vein diameter was calculated using the formula $D = \sqrt{a \times b}$, where a represents the major diameter and b represents the perpendicular minor diameter. By using this formula, the average diameters of all central veins drawn were found, and their arithmetic mean was calculated [38].

Standardization of Magnification, Field Selection, and Evaluation in Histopathological Scoring

For the evaluation of experimental liver injury models by histopathology, criteria for the magnification, the number of fields, and the selection of the fields have to be fixed before the evaluation. Semi-quantitative scoring can even increase the strength of the comparison between the study groups by transforming the qualitative findings into numbers. To fix the above-mentioned criteria for the evaluation, however, the same magnification for the evaluation has to be used as well as the same number of fields, and, if possible, the evaluation has to be performed blinded [8,9]. When examining liver sections, it is initially advisable to assess the overall architecture of the liver, the lobular structure, the extent of necrosis, the degree and distribution of fibrosis and steatosis using low magnification (x4 or x10). Subsequently, randomly selected fields are scored at the magnifications of x10, x20, or x40, depending on the parameter in question. For each animal, at least five fields should be evaluated in more detail, but 10 fields or more are preferred, in order to be able to assess inhomogeneous injury patterns. When scoring within a study group, the magnification, the number of fields, and the criteria used for scoring must be identical for all samples (Table 8) [1,2,6,7].

**Table 8:** Recommended magnification and number of microscopic fields for histopathological scoring of liver injury parameters. The table summarizes suggested magnifications, recommended numbers of fields, and evaluation criteria for the assessment of major histopathological parameters in experimental liver injury models. The table includes hepatocellular necrosis, inflammatory cell infiltration, hepatocyte degeneration and vacuolization, sinusoidal congestion and dilatation, steatosis and hepatocyte ballooning, cholestasis and bile duct proliferation, fibrosis and collagen deposition, and total histological damage score. These recommendations aim to support standardized field selection, reproducible scoring, and comparable histopathological evaluation across experimental groups. NAS: NAFLD Activity Score; NAFLD: non-alcoholic fatty liver disease

Scoring parameter	Suggested magnification	Number of suggested fields	Evaluation score
Hepatocellular necrosis	10x-20x	5-10 random areas per animal	The extent, localization, and severity of necrosis are assessed. A 10x magnification is suitable for large areas of necrosis, and 20x magnification for cellular detail.
Inflammatory cell infiltration	20x-40x	5-10 random areas per animal	Portal, lobular, or perinecrotic inflammation is assessed. A 40x magnification is preferred for cell density.
Hepatocyte degeneration and vacuolization	20x-40x	5-10 random areas per animal	Hepatocyte swelling, hydropic changes, cytoplasmic pallor, and vacuolization are evaluated.
Sinusoidal congestion and dilation	10x-20x	5-10 random areas per animal	Blood pooling, dilation, and impaired microcirculation in the sinusoids are evaluated.
Steatosis and hepatocyte ballooning	10x-20x; 20x-40x for ballooning	5-10 random areas per animal	The extent of steatosis is assessed with 10x-20x magnification, and ballooned hepatocytes with 20x-40x magnification. In the NAS system, steatosis, lobular inflammation, and ballooning are scored together [2].
Cholestasis and bile duct proliferation	10x-20x; 40x for cellular detail	5-10 portal areas per animal	Bile pigment accumulation, canalicular cholestasis, and ductal reaction are evaluated. Portal area selection is important.
Fibrosis and collagen deposition	4x-10x; 20x for detail	Entire section or 5-10 areas per animal	Fibrotic areas, septa, and bridging fibrosis are evaluated in Masson trichrome or Sirius red-stained sections. If digital analysis is to be performed, the entire section or standard areas can be used.
Total histological damage score	10x-40x according to the parameter	5-10 random areas per animal	This is achieved by individually scoring parameters such as necrosis, inflammation, degeneration, congestion, steatosis, or fibrosis, and then calculating the total score.

When selecting the fields for evaluation of a liver specimen, the choice of fields should be random; i.e., avoid fields with artifacts (e.g., tears in tissue, folds in sections, severe overstaining or loss of tissue), but rather choose fields that will accurately represent both the area of maximum injury and the normal, unaffected liver tissue. There are models of injury (e.g., APAP and CCl_4_ poisoning) that induce more heterogeneous injury, with involvement of the pericentral regions surrounding the central vein and varying degrees of injury to the surrounding parenchyma. Both should be taken into account.

Scoring should be performed, whenever possible, in a blinded manner by two independent evaluators, such as a researcher, histologist, or pathologist. In animal research using experimental models, blinding, randomization, and precise description of the experimental details are critical for making experiments as reproducible as possible [9]. All details of histological evaluation (magnification used, number of fields examined, whether fields were chosen randomly, range of score used, etc.) should be provided in a detailed description of the methods that were used.

As a rule of thumb for experiments on liver damage, it is recommended to assess at least 5 to 10 randomly selected fields per animal. To achieve reproducibility, it is essential to analyze all fields at the same magnification. Detailed examination of cells is best performed at x20 to x40 magnification, while assessment of larger areas of tissue and the distribution of lesions is carried out at x4 to x10 magnification. In this way, assessment of histological damage is standardized and thus becomes more objective, and results can be compared more easily.

In addition to standardization of magnification, field selection, and blinded evaluation, interobserver agreement is an important factor in the reliability of histopathological scoring. When semi-quantitative scoring systems are used, evaluation by more than one observer and assessment of concordance between observers may improve the reproducibility of the results. Validation metrics, including agreement-based or correlation-based analyses, can also support the consistency of scoring systems across different observers, laboratories, and experimental models. In recent years, digital pathology and AI-assisted image analysis have also emerged as supportive tools for reducing observer-related variability and improving quantitative tissue assessment. However, these approaches should be considered complementary to conventional histopathological scoring and should be interpreted within the context of the experimental model, staining method, image quality, and validation procedure.

Integration of Immune Cell Scoring Into Liver Histopathological Assessment

The semi-quantitative grading approach used in experimental liver histopathology can also be adapted to assess immune cell infiltration when specific inflammatory cell populations are identified with appropriate markers. Brzóska et al. evaluated liver and kidney histology in rats exposed to cadmium and ethanol and demonstrated that tissue changes in experimental toxicity can be assessed using severity-based histological grading [40]. While this study did not specifically score neutrophils, macrophages, or T lymphocytes as separate immune cell subsets, it supports the general principle that histopathological changes in rat liver tissue can be classified as absence, mild presence, moderate severity, or significant/severe involvement. Based on this approach, immune cell-specific assessment can be used to grade marker-positive neutrophils, macrophages, or T lymphocytes according to their density, distribution, and localization within damaged liver tissue.

Discussion

Tissue damage in experimental models of liver injury can be quantified by histopathological scoring of various parameters rather than simple descriptive microscopic examination. Scoring of histopathological changes has advantages over simple, observer-dependent qualitative histopathological assessment of injury. Semi-quantitative scoring parameters of tissue damage and inflammation enable strong correlation of lesions and qualitative and quantitative changes of experimental models of human liver disease. These changes are transferred to numerical values, which allow translations of results into human studies of comparable design and thus to results of human studies. The process of histopathological scoring is influenced by scoring criteria. The use of scoring criteria to define lesions as measured in semi-quantitative scoring systems to define parameters of experimental liver injury renders the assessment of histological damage, as used in experimental studies, more objective. Moreover, histopathological findings can be transformed into numbers by tissue scoring systems, which then allow for comparisons of degrees of experimental lesions of interest between experimental groups within study designs of experimental research [6]. For that reason, semi-quantitative scoring of tissue injury and/or inflammation is a very important parameter of histopathological findings, which are scored in an experimental study within the frame of pathology of experimental research in general and for qualitative findings in particular. Thus, multiparametric scoring systems that include quantitative histopathological assessment, as used for qualitative histopathological findings in animal models of human disease, are pivotal for experiments using animals to find translatable results [22].

Based on their relevance to evaluate various aspects of liver injury, parameters such as hepatocellular necrosis, numbers of inflammatory cells, hepatocellular changes, sinusoidal congestion, steatosis, cholestasis, and fibrosis are typically assessed and scored in respective experimental models of liver injury. For instance, the Suzuki score is a widely accepted semi-quantitative scoring system for evaluating the extent of histopathological changes, including sinusoidal congestion, hepatocyte vacuolization, and necrosis, that occur in hepatic ischemia-reperfusion injury. The NAS for assessing the degree of liver injury in NAFLD/NASH is another typical example of the combination of steatosis, lobular inflammation, and hepatocellular changes, including ballooning and cytological damage that are commonly assessed in experimental models of rodent NAFLD and subsequently scored in histopathological evaluations [2]. It is also worth noting that scores for chronic findings such as fibrosis and collagen deposits can be quantified using computerized image analysis after staining with Masson’s trichrome or Sirius red. For this purpose, the amounts of fibrogenic collagen in livers of rodent models of CCl₄-, TAA-, and BDL-induced chronic liver fibrosis were quantified by using computerized image analysis after staining with Masson’s trichrome and/or Sirius red [15].

Chronic injury of liver tissue, like fibrosis or collagen deposition, can even be analyzed by digital image analysis using tissue-specific staining like Masson’s trichrome or Sirius red. The scoring of histological parameters, even by semi-quantitative scoring systems, is enhanced by supporting the assessment with morphometric analysis, as Liu et al. did in a study in which they used CCl₄, TAA, and BDL to induce liver fibrosis in rodents. In this study, they verified the application of their novel concept by automated image analysis in all these models of chronic liver injury using corresponding established scoring systems and, furthermore, by additional novel scoring systems of fibrosis in these models [15]. In summary, morphometry as automated image analysis in liver pathology can enhance the quality of experimental studies on acute as well as chronic liver injury.

Histopathological scoring is very much dependent on the scoring system that is chosen to evaluate lesions of interest, but also on the method used to score them. By using the same magnification to examine samples and taking a random number of fields from each time, samples can be made more comparable. Furthermore, it is essential to avoid areas with artifacts and to blind samples for scoring purposes, where possible, to avoid influencing the results due to preconceived ideas of the expected findings. The Animal Research: Reporting of In Vivo Experiments (ARRIVE) 2.0 guidelines have recently highlighted the importance of randomization, blinding, and transparent reporting of study methods to allow for reproducibility of animal studies [1]. Histopathological assessment in combination with biochemistry, IHC, and molecular techniques can now provide a more complete picture of the type of liver injury that is induced in experimental models.

## Conclusions

Histopathological scoring of liver injury in experimental models has the advantage that qualitative observations can be transformed into quantitative data. The parameters to be scored, however, are not always of equal importance in different models and therefore have to be chosen on the basis of the respective model used, the aim of the study, and the type of liver injury that is induced. Therefore, histopathological scoring is a reliable method for evaluating experimental liver injury, provided that appropriate magnification is used, a sufficient number of fields are examined, and blinded assessment is performed.
